# Building a sustainable management pipeline: Key predictors of nurse management aspirations in Ireland

**DOI:** 10.1371/journal.pone.0355484

**Published:** 2026-08-07

**Authors:** Elaine Berkery, Nuala F. Ryan, Helen Purtill

**Affiliations:** 1 Department of Management and Marketing, Kemmy Business School, University of Limerick, Limerick, Ireland; 2 Department of Mathematics and Statistics, Faculty of Science and Engineering, University of Limerick, Limerick, Ireland; Guangxi Normal University, CHINA

## Abstract

**Background:**

Against the backdrop of the shortages of nursing managers, this paper examines predictors of career aspirations among nurses in Ireland. Rather than viewing career aspirations as being determined solely by personal, job related or organizational factors we consider the individual nurse, within the context of their job, interacting with the overall organization and seek to ascertain how these factors are related to nurse’s career aspirations.

**Objective:**

The objective of this study is to explore how personal, job-related, and organizational factors shape nurses’ career aspirations. The research question guiding this study is: “What variables relating to the individual, job or organization predict career aspirations among nurses?”.

**Design:**

This cross-sectional study gathered questionnaire data from 263 nurses.

**Setting:**

A large public hospital group in Ireland comprising 5 hospitals.

**Methods:**

Pearson correlations provided an initial understanding of the bivariate relationships between scales measuring career aspirations and scales measuring resilience, job satisfaction, employee engagement, psychological safety, organizational citizenship behavior, and organizational commitment. Multivariate Linear Regression models examined factors independently associated with career aspirations.

**Results:**

Correlational analysis identified statistically significant relationships between career aspirations and the individual, job and organizational scales, with resilience showing the strongest positive correlation (r = 0.418, p < 0.01). The Multivariate Linear Regression analysis identified having a postgraduate qualification (β = 2.92, 95% CI [1.38, 4.45], p < 0.001) and higher levels of resilience (β = 2.22, 95% CI [1.26, 3.19], p < 0.001) as statistically significant independent predictors of career aspirations.

**Conclusion:**

The findings provide insights into developing strategies to create a pipeline of qualified nurses to optimize recruitment and succession planning for nurse managers, thereby building a sustainable workforce. Hospital managers should develop policies and programs that promote organizational support for further education and resilience-building workshops. These initiatives could encourage more nurses to pursue nursing management roles.

## Introduction

The global shortage of nurse managers poses significant challenges to healthcare systems. Nurse managers are crucial in ensuring the smooth operation of healthcare facilities, mentoring staff, and maintaining high standards of patient care [1]. Nurse managers not only play a critical role in healthcare by preventing adverse patient outcomes, support patient recovery, and enhancing overall satisfaction with medical services [2], they are vital to achieving Sustainable Development Goals pertaining to health (Sustainable Development Goal 3), as well as Sustainable Development Goals pertaining to gender equality (Sustainable Development Goal 5), education (Sustainable Development Goal 4), and the pursuit of decent employment and economic growth (Sustainable Development Goal 8) [3]. However, the global nursing sector faces significant staffing shortages, estimated to be at 4.5 million [4], which includes a sizable shortage of nursing managers [5,6]. The shortage of nurse managers is mainly owing to the fact that current nurse managers, who are in the baby boomers generation, are retiring [5], and career aspirations among nurses to managerial roles can be low [6,7]. The literature also reports that few staff nurses are interested in seeking a future in nurse manager roles [8,9]. Within an Irish context, while international nurses are heavily relied upon to combat current nurse shortages, Humphries et al [10] highlight that only a few international nurses had advanced into managerial grades, noting that perhaps this had stemmed from their reluctance to apply for such roles. These trends are worrying given the crucial role career aspirations can play as antecedents for developing a sustainable workforce [11].

Policy documents, such as the International Council of Nurses’ “Charter for Change” [1] emphasize strategies to recruit, retain, and develop nurses to help overcome the current shortage of nurses. The key points within this document which are relevant to this study include creating growth opportunities, retaining current staff, and supporting career progression. While a significant amount of work has been done on developing career pathways for the nursing profession, both professionally [12,13] and within the literature [14,15], there is a lack of information on what influences nurses career aspirations to become managers [7,16]. The limited research that is available has identified personal factors as being more strongly associated with career aspirations than situational factors, however, very few studies conducted within the nursing profession have specifically examined factors influencing career aspirations to management roles [16]. Furthermore, previous studies have overlooked the relationship between the individual nurse, their job, and the broader organizational contexts, focusing primarily on personal factors [16]. As a result, little is known about how individual and intrinsic factors arising from the job and workplace influence career aspirations. To address this gap, we apply a multi-factor perspective to the examination of predictors of career aspirations among nurses and seek to answer the following question: “What variables relating to the individual, job or organization predict career aspirations among nurses?”

## Overview of literature

Drawing from studies across the nursing literature, and wider management literature, we have identified personal factors influencing career aspirations, as well as intrinsic related factors arising from the job itself (job related variables and organizational factors) that can influence career aspirations. Hereafter we summarize what is currently known about individual, job related, and organizational factors and their relationship with career aspirations.

Individual factors such as age, educational level, length of service, domestic nurses/international nurses, and individual resilience have all been shown to be possible predictors of career aspirations among nurses [16,17]. Studies to date have found that younger nurses are more interested in pursuing management roles in nursing [7,16], with a lack of appropriate university qualifications being cited as a possible factor leading to lower career aspirations among older nurses [16]. Furthermore, as healthcare organizations rely heavily on international nurses to fulfil vacancies, it is important to understand the career aspirations of international nurses. While studies have shown that a common driver for nurse migration is career aspirations [17], little is known about the similarities and differences of career aspirations between domestic and international nurses in the same healthcare setting. Finally, resilience, which is defined as “an individual's ability to thrive despite adversity” [18], taking into account an individual’s ability to cope with stress [19]. Studies have shown a positive relationship between resilience and career aspirations [20]. Resilience enables workers to adapt to dynamic work environments, contributing to enhanced job satisfaction [21] and commitment to the profession [22], particularly in cases of failed promotion attempts where resilient nurses are better equipped to recover quickly, allowing them to maintain focus on long-term career goals [23].

Moving to intrinsic factors, job related and organizational factors. Job satisfaction reflects how individuals feel about their work, influencing their career aspirations [24]. For instance, a recent study of millennial nurses in the United States showed a significant connection between job satisfaction and leadership aspirations [5]. However, and counterintuitively, findings from an older study conducted among an American cohort of nurses found that nurses who intended to stay at the same organization in the same position had significantly higher levels of job satisfaction, and in many cases nurses with the highest levels of job satisfaction reported that they intended to leave nursing within the next 5-years [25]. Employee engagement has also shown to be a key ingredient in organizational efforts to retain nurses [26]. When employees are engaged, they have a stronger sense of purpose and connection with their work and are more likely to be proactive about their career [27–29]. Lastly in terms of job related factors, psychological safety, which can be defined as an individual’s perception of interpersonal risks [30], and the shared belief that individuals can express ideas and concerns without fear of negative consequences [31], is particularly important in healthcare organizations as it helps to ensure patient safety and high quality care [32]. Studies to date have linked psychological safety to organizational commitment [33] and employee engagement [34], however the impact of psychological safety on career aspirations remains unexplored, highlighting the need for further research. Finally, turning to organizational factors, studies to date have shown how organizational factors such as organizational commitment and organizational citizenship can impact career aspirations. Committed nurses are more motivated to pursue professional growth, while those less committed are more likely to leave their roles [35]. Similarly, organizational citizenship behavior, which refers to contributions beyond formal requirements [36], plays a crucial role in improving patient outcomes [37] and career advancement [38,39]. Employees with high organizational citizenship behavior are perceived as more valuable, leading to better appraisals and opportunities for progression [38,39].

Taken as a whole, and as evidenced in the literature above, there are a plethora of factors that can influence career aspirations in healthcare settings, although the relationship between the factors remains underexplored. The objective of this study is to consider the individual nurse, within the context of their job, within the overall organization, and seek to ascertain how these factors work together to influence nurse’s career aspirations. The research question guiding this research is as follows: “What variables relating to the individual, job or organization predict career aspirations among nurses?”

**Fig 1:**
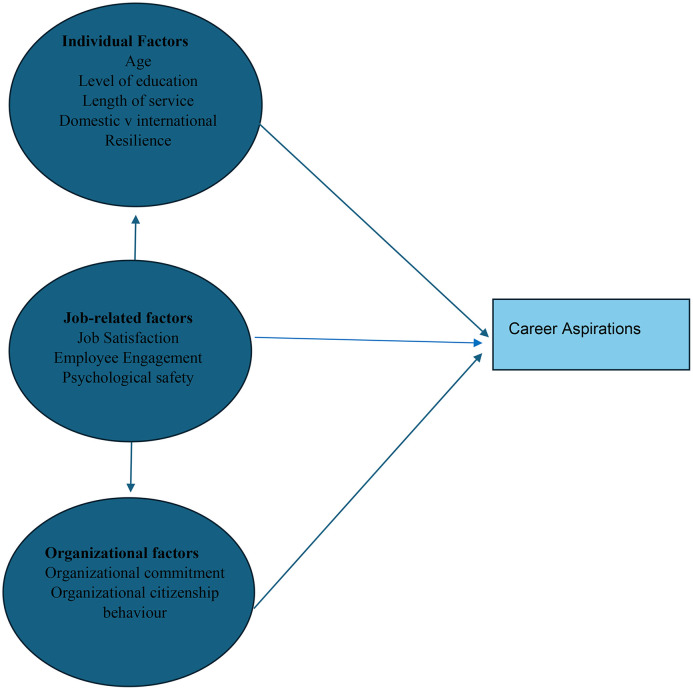
Study Framework: Possible individual, job and organizational predictors of career aspirations.

The study framework used in this study is outlined in Fig 1, illustrating the individual, job related and organizational factors used in our analysis to determine predictors of career aspirations among nurses.

In the next section we outline the methods and procedures used in this research.

## Materials and methods

### Study design

This cross-sectional study was conducted across a large hospital group in Ireland that serves a population of 380,000 people.

### Study setting

The hospital group, which is partnered with a local university, comprises six hospitals in total, one Model 4 hospital (bed capacity 535 inpatient beds), two Model 2 hospitals (bed capacity 50 inpatient beds and 52 inpatient beds respectively), one Model 2-S hospital (bed capacity 89 inpatient beds), and two specialty hospitals (one maternity, bed capacity 83 and 19 cots in Neonatal and one orthopedic, bed capacity 44 inpatient beds). The study took place across five individual sites: one Model 4 hospital, two Model 2 hospitals, one Model 2-S hospital and one specialty hospital (orthopedic). All nurses working across the hospital group were invited to participate in the study. At the time of data collection there were 2,040 whole time equivalent (WTE) nurses and midwives employed within the hospital group [40]. Please note that all Health Service Executive (HSE) (Ireland’s public health system, created under the Health Act 2004 to bring hospitals, community services, and social care under one national structure) workforce publications combine nurses and midwives into a single category. Data for this study was only collected from the nursing cohort.

### Variables

The data collected in this study included both demographic variables (such as age, level of employment, length of service within the hospital group, country where their initial nursing qualification was obtained) and preexisting scales which were used to enhance the reliability and validity of these findings.

### Demographic characteristics

Data on respondents age, length of service, educational level, and whether the nurse was an international or domestic nurse was collected.

### Career aspirations

O’ Brien’s [41] Career Aspiration Scale was used in this research as the dependent variable. The scale comprises 8 items on a 5-point Likert-type scale. Participants were asked to indicate how true each statement is for them on a scale ranging from 1 (not at all true of me) to 5 (very true of me). Four items are reverse scored, and items are summed to obtain a total score, with a higher score indicating greater aspiration within a given career.

The scales for predictor variables included:

### Resilience

Connor-Davidson Resilience Scale 10 item scale five-point Likert scale (from 1 “not at all descriptive,” to 5 “very descriptive”) (CD-RISC-10) developed by Campbell-Sills and Stein [18] was used to measure an individual’s resilience indicating how well equipped one is to bounce back after stressful events, tragedy, or trauma that can arise throughout one’s life.

### Employee engagement, job satisfaction and affective organizational commitment

In measuring Happiness at Work, the shortened form of Salas-Vallina and Alegre (42) happiness at work scale was used to measure employee engagement (3 items), job satisfaction (3 items), and affective organizational commitment (3 items) using a 5-point Likert scale for each item. On these scales, higher scores indicate, higher levels of engagement, satisfaction and organizational commitment [42].

### Psychological safety

Edmondson's (1999) seven-item five-point Likert scale (from 1 “not at all descriptive,” to 5 “very descriptive”) is the most widely used indicator of psychological safety. The questions are designed to gauge a person's belief that they can take risks with their team [30].

#### Organizational citizenship behavior.

We assessed organizational citizenship behavior directed towards individuals using Lee and Allen’s [43] eight item 5-point Likert scale from 1 “not at all descriptive” to 5 “very descriptive”.

### Ethical approval

This study was approved by the ethics and research committees of the hospital group (REC REF:104/2021) and home university (KBS Research Ethics Application: 2021_12_KBS_15). All procedures performed in this study involving human participants were conducted in accordance with the ethical standards of the hospital groups and home university research committee and with the 1964 Helsinki Declaration ethical standards. Participants were provided with a subject information sheet at the start of the questionnaire, outlining details of the project. Prior to completing the questionnaire participations were provided with an information sheet outlining the purpose of the study, their right to withdraw at any stage without penalty, the opportunity to ask questions prior to participating in the study, and assurance that information provided would remain confidential and anonymous. Participants were then asked for consent to participate in the study by ticking “yes” or “no” to the following statement “I agree to give consent”. Each response was anonymized using a number key to maintain confidentiality.

### Data collection

Data collection took place between 12/09/2022 and 14/10/2022. Both hard and soft copies of the questionnaire were distributed. To inform nurses about the study, posters and flyers with a QR code to access the questionnaire were distributed across the 5 sites, details of the questionnaire were included in the Hospital Groups Newsletter for Nurses, Midwives and Health Care Assistants, and finally, nurses were contacted via internal email detailing the research and a link to the online questionnaire. The research team had a physical presence across the five sites during the roll out of the questionnaire to inform nurses of the study and to manually distribute questionnaires for completion.

Sample size considerations were based on recommended guidelines of at least 10–20 observations per predictor variable for the planned analyses [44] and a sample size of 260 met these requirements. Additionally, it provided adequate power (≥80%) to detect small-to-moderate effect sizes at a significance level of 0.05.

### Data measurement and statistical methods

All analyses were undertaken using IBM SPSS V29, using a 5% level of significance throughout. Descriptive statistics summarize categorical variables as number (percentage). Cronbach’s alpha was used to examine the internal reliability of the scales. Pearson correlations summarized the bivariate associations between career aspiration and the scales measuring individual, job related and organizational factors. Multiple linear regression was used to develop the three models for career aspiration informed by the study framework (Fig 1). All numeric predictor variables were standardized prior to inclusion in the regression models, and results are reported as standardized regression coefficients (β) with corresponding 95% confidence intervals. Multicollinearity was assessed using variance inflation factors (VIFs). All included variables had VIF values below 5, indicating that multicollinearity was not a concern in the regression models. Model assumptions were assessed using residual diagnostic plots, including a histogram of the residuals and a scatterplot of predicted values versus standardized residuals. These diagnostics found no evidence of outliers or departures from the assumptions of normality or homoscedasticity. Regression analyses were conducted using listwise deletion, such that cases with missing data on any variable included in the model were excluded. The scales used in our questionnaire are preexisting scales, which have been widely used and tested within the literature. The reliability of each scale is outlined in Table 1. Lower Cronbach’s alpha values for some scales (job satisfaction and employee engagement) may be attributable to their limited number of items, given the influence of scale length on internal consistency. The psychological safety measure also yielded an alpha below 0.70; however, similar variability was reported by Rathert et al. [45] when using a shortened 6-item version of Edmondson's scale. Nonetheless we acknowledge these scores are below the commonly cited α = 0.70 criterion and acknowledge this as a limitation to our study and caution the interpretation of these results accordingly. This is discussed in greater detail in the limitations section.

**Table 1:** Reliability statistics for scales.

Scale	Cronbach Alpha	Number of Items	Range of Scale	n
Career aspirations	0.79	8	8 - 40	263
Resilience	0.86	10	10–50	261
Job Satisfaction	0.66	3	1 - 5	263
Psychological safety	0.67	7	7–35	262
Employee engagement	0.65	3	1–5	263
Organization citizen behavior	0.86	8	8–40	261
Organizational commitment	0.89	3	1–5	263

### Potential bias

To mitigate the effects of common method bias several steps were taken. Anonymity was assured to all respondents, doing so reduced the respondents urge to provide socially desirable, expected or accommodating answers [46]. Furthermore, independent and dependent variables were placed in different sections of the questionnaire, decreasing the likelihood that respondents would artificially answer each question in the same way [46].

## Results

A total of 263 responses were collected, of which 222 were online responses and 41 were collected by hard copy. This represents an overall response rate of 12.9%. As it was not possible to separate the number of nurses from the total number of nurses and midwives employed in the group, the response rate reported of 12.9% is taken from the total WTE figure of 2,040 nurses and midwives employed at the time of surveying. The descriptive characteristics of respondents are outlined in Table 2. The proportion of missing data was low (<1%) across all variables. 46.8% of respondents had previously applied for promotion within the hospital group, resulting in an 80.5% success rate for applicants. In the next section we examine the relationships between the different variables and career aspirations.

**Table 2:** Descriptive characteristics of respondents.

Gender	Male Female Non-binary I would prefer not to disclose	16 (6.1%) 244 (92.8%) 0 (0.0%) 3 (1.1%)
**Age**	18 - 30 31 - 40 41 - 50 51+	48 (18.3%) 95 (36.1%) 73 (27.8%) 47 (17.9%)
**Staff Nurse**	No Yes	102 (38.5%) 161 (61.5%)
**International nurse or Irish nurse**	International Irish	96 (36.6%) 166 (63.4%)
**Years service with hospital group**	0-5 years 6-15 years > 15 years	133 (50.8%) 43 (16.4%) 86 (32.8%)
**Postgraduate Qualification Completed**	Yes No	125 (47.9%) 136 (52.1%)

Pearson correlation values for the career aspirations with the potential predictor variables included in the regression models (standardized to z-scores) are presented in Table 3.

**Table 3:** Pearson correlations.

	n	M (SD)	1	2	3	4	5	6	7
**1. Career Aspiration**	263	28.79 (6.64)	--						
**2. Resilience**	261	38.44 (6.50)	.417**	--					
**3. Job Satisfaction**	263	3.34 (.81)	.189**	.209**	--				
**4. Psychological Safety**	262	21.03 (4.84)	.157*	.126*	.586**	--			
**5. Employee Engagement**	263	3.53 (.85)	.331**	.336**	.446**	.421**	--		
**6. Organizational citizenship behavior**	261	32.46 (5.32)	.320**	.494**	0.101	.171**	.436**	--	
**7. Organizational Commitment**	263	2.94 (1.24)	.172**	.245**	.624**	.519**	.528**	.226**	--

** Correlation is statistically significant at the 0.01 level (2-tailed).

* Correlation is statistically significant at the 0.05 level (2-tailed).

Pearson’s correlations identify statistically significant relationships between career aspirations and resilience, job satisfaction, psychological safety, employee engagement, organizational citizenship behavior, and organizational commitment. Among these, resilience (.417, p < 0.01) showed the strongest positive correlation with career aspirations. Multiple linear regression was used to fit three models to the data: Model 1- Individual factors, Model 2– Job related variables, controlling for individual factors, and Model 3- Organizational factors, controlling for individual and job-related factors. These results are outlined in Table 4.

**Table 4:** Multiple linear regression analysis of career aspirations.

	Model 1		Model 2		Model 3	
Coeff. (95% CI)	p-value	Coeff. (95% CI)	p-value	Coeff. (95% CI)	p-value
Intercept	25.26 (23.3, 27.22)	<0.001	25.35 (23.42, 27.28)	<0.001	25.21 (23.27, 27.15)	<0.001
**Age** (ref 51 + years) 18-30 years 31-40 years 41 - 50 years	−0.06 (−3.04, 2.91) 1.57 (−1.02, 4.16) 0.95 (−1.31, 3.2)	0.967 0.234 0.408	−0.33 (−3.26, 2.61) 1.1 (−1.46, 3.66) 0.73 (−1.51, 2.96)	.827 .398 .522	0.01 (−2.97, 2.99) 1.32 (−1.26, 3.9) 0.84 (−1.4, 3.07)	.996 .313 .462
**Education** (postgraduate)	2.84 (1.3, 4.38)	**<0.001**	2.79 (1.27, 4.3)	**<0.001**	2.92 (1.38, 4.45)	**<0.001**
**Domestic v international** (ref. Domestic)	0.93 (−1.09, 2.94)	.365	−0.17 (−2.24, 1.91)	0.875	0.26 (−1.86, 2.39)	0.807
**Length of service** (ref. > 15 years) 0 - 5 years 6–15 years	1.58 (−0.93, 4.08) 0.34 (−2.12, 2.8)	0.216 0.785	2.58 (0.06, 5.1) 1.33 (−1.15, 3.8)	0.045 0.292	2.16 (−0.44, 4.75) 1.27 (−1.22, 3.75)	.103 .317
**Resilience**	3.07 (2.24, 3.89)	**<0.001**	2.55 (1.69, 3.41)	**<0.001**	2.22 (1.26, 3.19)	**<0.001**
**Satisfaction**			0.47 (−0.47, 1.41)	0.324	0.7 (−0.34, 1.73)	.184
**Psychological Safety**			0.25 (−0.68, 1.18)	0.593	0.21 (−0.75, 1.16)	.671
**Employee Engagement**			1.09 (0.21, 1.97)	**0.016**	0.85 (−0.12, 1.83)	.087
**Organizational Citizen Behavior**					0.85 (−0.06, 1.76)	.067
**Organizational Commitment**					−0.25 (−1.28, 0.78)	.63
Model Fit (R-square)	0.235 (n = 260)		0.275 (n = 258)		0.273 (n = 257)	

^*1*^ *Scales were standardized to z-values prior to fitting the regression model.*

^*2*^ *Coefficient*

^*3*^ *Standard Error*

The results from Model 1 indicate that completion of a postgraduate qualification (β = 2.84, 95% CI [1.3, 4.38], p < 0.001) and higher levels of resilience (β = 3.07, 95% CI [2.24, 3.89], p < 0.001) were statistically significantly associated with higher career aspirations. Age, domestic versus international, and length of service were not statistically significant in predicting career aspirations. Within Model 2, which examined job-related factors controlling individual level factors, completion of a postgraduate qualification (β = 2.79, 95% CI [1.27, 4.3], p < 0.001), higher resilience (β = 2.55, 95% CI [1.69, 3.41], p < 0.001) and higher employee engagement (β = 1.09, 95% CI [0.21, 1.97], p = 0.016) were independent predictors of higher career aspirations. Similarly, within Model 3, which included individual factors, job-related factors and organizational factors, completion of a postgraduate qualification (β = 2.92, 95% CI [1.38, 4.45], p < 0.001) and higher resilience (β = 2.22, 95% CI [1.26, 3.19], p < 0.001) remained statistically significant independent predictors of higher career aspirations, controlling for all other variables in the model.

## Discussion

The overall aim of this study was to identify specific predictor variables at individual, job-related, and organizational level that influence career aspirations among nurses. To do so, we adopted a multi-factor perspective in our analysis to set about answering the following research question “What specific variables at individual, job-related, or organizational levels predict career aspirations among nurses?” This allowed us to consider individual factors related to nurses, working in their roles, within an organization, and examine how the three layers influence career aspirations. From an individual perspective the completion of a postgraduate qualification and higher levels of resilience were statistically significantly associated with higher career aspirations. Turning to job-related factors, higher employee engagement was an independent predictor of higher career aspirations, however, after controlling for variables at all levels, employee engagement was no longer a predictor of career aspirations. Finally, at the organizational level, no factors were statistically significantly related to career aspirations. The overall findings of this study indicate that when controlling all factors (see Model 3), the completion of a postgraduate qualification and higher resilience were the only variables statistically significantly associated with higher career aspirations.

### Theoretical implications

From a theoretical perspective, studies to date have identified individual factors that can impact career aspirations. However, these variables were primarily examined in isolation, and as a result fail to identify whether other factors from the individual, job, or organization were likely to have an impact on career aspirations, as evidenced in this study. At a bivariate level, Pearson’s correlations identify statistically significant relationships between career aspirations and resilience, job satisfaction, psychological safety, employee engagement, organizational citizenship behavior, and organizational commitment. However, the findings from our multiple linear regression show that when individual level, job related, and organizational variables were controlled for, only resilience and the completion of a postgraduate qualification were statistically significant predictors of career aspirations. This finding can be explained by the fact that organizations are multifaceted and as such every outcome is tied to multiple organizational layers such as individuals, job, and the overall organization [47]. It is therefore important that any future research on predictors of career aspirations takes into consideration the interplay of factors across multiple domains such as the individual, their job, and the organization to unpack the underlying predictors of career aspirations.

### Practical implications

From a practical perspective, the shortage of nurse managers has been clearly documented within the literature [5,6], as well as the need to build a strong and sustainable workforce [1]. The findings from this study highlight the need to promote continuous learning and professional development, to build a more educated and resilient workforce, which in turn should lead to a workforce with higher career aspirations. Furthermore, the future of nursing and nursing managers is depended on robust succession planning [6]. Due to the impending shortage of nurse managers [48] organizations need to invest in education so that (1) talented and knowledgeable candidates are available to fill these roles, and (2) based on the results of this study, to develop a cohort of internal candidates interested in applying for promotional opportunities. Interestingly, and within the context of the findings from this study, studies to date have shown that nurses with higher educational attainment show higher workplace resilience [21,49]. While there is an established line of enquiry within the nursing literature linking resilience to job satisfaction, self-esteem, self-efficacy, enhanced patient satisfaction, and favorable patient outcomes, and negatively correlating resilience with depression, anxiety, stress, burnout and turnover intention [21,49,50], the findings from this study add to this literature creating a further link between resilience and career aspirations. The Harvard Business Review define a resilient workforce as “a group of employees who not only are dedicated to the idea of continuous learning but also stand ready to reinvent themselves to keep pace with change; who take responsibility for their own career management; and, last but not least, who are committed to the company’s success” [51]. Within this context, each employee must recognize the key skills and behaviors their employer will require for future roles, as well as being aware of one’s own skills and knowledge, to ensure they have the necessary skills to adapt to future roles that will help them progress their career.

### Managerial implications

From a management perspective, a recent meta synthesis of lifelong learning and nurses’ continuing professional development recommended that access to continuing professional development within the nursing profession should be made more attainable, realistic and relevant [52], with a need to ensure more funding and accessibility to nurses to engage in professional development. From the perspective of nurse managers shortages, continued professional development initiatives can contribute to both the attraction and attainment of nurses [52,53]. However, it is critical that the organizational culture is one that supports and encourages continued professional development [54]. For example, flexible off-duty patterns should allow time for nurses to study, financial support to cover the cost of attaining a postgraduate qualification, and practical support from the organization in the form of adequate time to participate in further education, as well as arranging suitable staff cover when colleagues are away engaging in further education [52]. Those in nursing management roles should encourage and mentor staff that do not have postgraduate qualifications to pursue further qualifications. Nurses should not be deferred from pursuing postgraduate qualifications due to barriers outside of their control, such as poor staffing levels, heavy workloads, lack of funding, and lack of study time [52].

Healthcare organizations ought to engage in, and promote, resilience training programs for their staff. Organizations should host courses, seminars, conferences and workshops that enable nurses to develop strategies to become more resilient. For example, workshops could be organized that enable nurses to express their concerns, share their workplace experiences and problems, and work together to develop methods to alleviate these concerns and problems [55]. Developing a resilient workforce will have positive effects on nurses’ attitudes towards the profession, their future within the profession, and their work outputs [55].

### Limitations

The following limitations should be taken into consideration when interpreting the findings of this study. Firstly, while the findings of this study are relevant to the hospital group in which the study was conducted, these findings may not be generalizable across all healthcare settings, therefore we recommend replica studies be carried out across multiple healthcare settings to determine generalizability. The moderate R² values indicate that the models explain a meaningful but incomplete proportion of variance in the outcomes, suggesting that additional unmeasured factors may be relevant. Although residual plots were examined to assess model assumptions, reliance on visual inspection may introduce subjectivity and reduce the sensitivity for detecting minor deviations from assumptions. Some scales demonstrated lower internal consistency, which may have attenuated observed associations. Measurement errors arising from lower reliability may weaken the strength of some of the relationships recorded in our regression analysis. Future studies would benefit from using full scales for job satisfaction and employee engagement as the shortened version of these scales may have limited capacity to achieve internal consistency. In addition, the cross-sectional design limits the ability to establish temporal relationships or draw causal inferences. With these limitations in mind, the methods employed in this study provide new insights into factors which influence nurses’ career aspirations to become nurse managers. Secondly, with a response rate of 12.9% the study is subject to potential non-response and self-selection bias, meaning the views of respondents may not fully represent the wider nursing workforce. Therefore, the findings from this study should be interpreted in the context of the response profile of respondents. Thirdly, the information gathered was self-reporting; yet, due to the nature of the data, no other method could have been used to get this amount of information. To reduce the impact of common method bias, several measures were implemented as outlined earlier in our potential bias section. To avoid common process variance, respondents were guaranteed anonymity. Finally, the results of this study are quantitative in nature. Follow up focus groups or interviews would enrich the data by providing a narrative to the results recorded here. Furthermore, focus groups with international nurses would be useful to capture their career aspirations as the Irish healthcare sector relies heavily on international nurses to overcome current shortages. This is against the backdrop of previous studies [10] that have indicated only a few international nurses had advanced into managerial grades, noting that perhaps this had stemmed from their reluctance to apply for such roles. However, our findings show that being an international nurse does not influence career aspirations. Future studies could unearth the reasons behind the low rate of international nurses in managerial roles.

## Conclusion

The overall findings of this study first highlight the multifaceted nature of organizations, illustrating how multiple organizational layers, and in this case how factors at the individual, job, and the overall organization level may predict career aspirations. Furthermore, the findings indicate how investing in continued professional development, focusing on the pursual of a postgraduate qualification and individual resilience building will enhance the nursing profession, helping to build a pipeline of suitably qualified and equipped nurses, that will not only fill future nursing management roles and alleviate the current shortage of nursing managers, but also build a more sustainable workforce. From a wider perspective, doing so will advance the Sustainable Development Goal pertaining to health (Sustainable Development Goal 3), as well as Sustainable Development Goals pertaining education (Sustainable Development Goal 4), and the pursuit of decent employment and economic growth (Sustainable Development Goal 8) [3].
